# Life-Cycle Switching and Coexistence of Species with No Niche Differentiation

**DOI:** 10.1371/journal.pone.0020314

**Published:** 2011-05-20

**Authors:** Javier Montero-Pau, Manuel Serra

**Affiliations:** Institut Cavanilles de Biodiversitat i Biologia Evolutiva, Universitat de València, Valencia, Spain; Hungarian Academy of Sciences, Hungary

## Abstract

The increasing evidence of coexistence of cryptic species with no recognized niche differentiation has called attention to mechanisms reducing competition that are not based on niche-differentiation. Only sex-based mechanisms have been shown to create the negative feedback needed for stable coexistence of competitors with completely overlapping niches. Here we show that density-dependent sexual and diapause investment can mediate coexistence of facultative sexual species having identical niches. We modelled the dynamics of two competing cyclical parthenogens with species-specific density-dependent sexual and diapause investment and either equal or different competitive abilities. We show that investment in sexual reproduction creates an opportunity for other species to invade and become established. This may happen even if the invading species is an inferior competitor. Our results suggests a previously unnoticed mechanism for species coexistence and can be extended to other facultative sexual species and species investing in diapause where similar density-dependent life-history switches could act to promote coexistence.

## Introduction

Maintenance of species diversity is a central topic in ecology, a critical issue being the limiting similarity of competing species allowing coexistence [1]. Most of the mechanisms that have been proposed to allow stable coexistence rely on niche differentiation (i.e. resource partitioning, differential vulnerability to predation, or differential response to temporal fluctuation or spatial variation). However, the number of cryptic species reported has dramatically increased since the introduction of the molecular techniques [2], and these species are frequently found in sympatry [3]–[5]. In many cases cryptic species do not show any clear niche differentiation, thus long-term co-occurrence of cryptic species may indicate that stable persistence of ecologically equivalent species is possible [6]. Therefore, explanations other than neutrality (i.e., lasting unstable coexistence) will be needed, and mechanisms able to explain stable coexistence not based on niche differentiation may be required.

A necessary condition for stable coexistence is population growth from low densities (i.e. invasibility criterion) [7]. For this to happen, the species with the highest density should affect their own growth more negatively than that of the rare species. Zhang and Hanski [8] showed that negative feedback could arise through features of sexual reproduction and recognized three mechanisms that could promote stable coexistence of identical competitors: density-dependent adjustment of sex ratio, sexual conflict, and sexually transmitted diseases. We propose another mechanism for stable coexistence of ecologically equivalent species based on density-dependent investment in sexual reproduction and/or dormancy. This mechanism may be especially important in facultatively sexual species (e.g., cyclical parthenogens), and species with diapause stages.

Sexual reproduction and diapause both impart a cost on population growth. Sexual reproduction typically incurs the “two-fold cost of males”, while diapausing stages usually exhibit a delay in hatching/germinating, so that part of the resources allocated to their production is lost to current population growth and competition efficiency [9]. This cost of sex and diapause could provide an opportunity for ecologically equivalent species making a lower investment in sexual reproduction or diapause to invade an assemblage. Hence, if sexual reproduction or diapause investment is density-dependent and controlled by species-specific signals a separate density-dependence occurs. It might create the negative feedback necessary for coexistence, even between species with otherwise completely overlapping niches. By investing in sex or diapause, a high-density competitor would decrease its own population growth rate more than that of its low-density competitor, allowing the rare competitor to grow faster. This could be the case for obligate sexuals, like the copepod species of the cryptic complex *Eurytemora affinis*, where crowding is the signal to produce dormant stages [10]; or for obligate asexuals, like *Bacillus* species where sporulation is triggered, among other cues, by quorum sensing [11]. Also, such a mechanism might be relevant for the coexistence of facultative sexuals, such as plants with a density-dependent switching between vegetative and sexual reproduction [12], [13] and cyclical parthenogens, where the costs of sex and dormancy are common. Cyclical parthenogenesis is a reproductive mode shared by approximately 15000 species [14] and is characteristic of aphids and two common zooplanktonic taxa –cladocerans and monogonont rotifers. This life cycle combines an extended phase of exclusive asexual (parthenogenetic) reproduction alternating with a phase of combined asexual reproduction and sexual reproduction, the latter leading to the production of diapausing stages. Sexual reproduction is known to be induced, among other clues, by population density in several groups of cyclical parthenogens [15], [16], and in at least three rotifer genera (*Epiphanes*, *Rhinoglena* and *Brachionus*) it is exclusively induced by population density [17]. One of the best-known mechanisms of sex induction is that of the rotifer *Brachionus plicatilis* species complex, where sexual reproduction is induced by a protein released into the environment by the rotifers [18]. As population density increases, this protein accumulates, and at a threshold concentration it triggers sexual reproduction, in a process akin to quorum sensing in bacteria [19]. Recently, it has been shown that some degree of specificity exists among these species regarding the induction of sex [20].

Here, we address the hypothesis that a density-dependent life cycle switch like the asexual to sexual transition can promote coexistence of otherwise ecologically equivalent species. Using the cryptic species complex *Brachions plicatilis* as a model, we develop and analyze a simple Lotka-Volterra competition model describing the dynamics of two competing species with a density-dependent investment in sex. We explore whether coexistence is possible in the extreme case of complete niche overlap between the competing species, first by assuming that density-dependent investment is exclusively dependent on conspecific density, and later by including the heterospecific density. We also explore the consequences of equal and unequal competitive ability between species.

## Methods

### Model

A modification of the model proposed by Serra and King [9] was used to describe the dynamics of the asexual (A_i_) and sexual (S_i_) individual densities for two competing species (i = 1, 2) having identical niches:
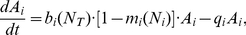
(1a)

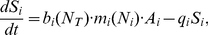
(1b)where b_i_(N_T_) is the birth rate of species i at total density N_T_ (N_T_ = Σ_i_ [A_i_+S_i_]), q_i_ is the mortality rate, assumed to be density-independent, and m_i_(N_i_) (where N_i_ = A_i_+S_i_) is the proportion of sexual individuals in the offspring of an asexual individual and is a measure of sexual investment. This proportion is assumed to be dependent on the species-specific density following a non-decreasing function. A species-specific dependence is a critical assumption which will discussed below. The exact definition of a sexual individual depends on details of the life cycle. For instance, in monogonont rotifers density of sexual individuals (S_i_) refers exclusively to sexual females since males are short-lived and do not feed, while in cladocerans and aphids it refers to sexual females (i.e., those producing haploid eggs) and males. Notice that S does not contribute births to the dA/dt or dS/dt of the current population because sexual reproduction is assumed to produce diapausing stages, and the model focus on the dynamics of the active stages. However, diapausing eggs matter for the long-term coexistence, and these implications will be discussed below. We assume a functional equivalence of sexual and asexual individuals except for their reproductive mode. Thus, birth rate and mortality rate are assumed to be equal for both types of individuals. Notice that, contrasting with the output of sexual reproduction –.i.e., diapausing eggs–, sexual individuals are active, consume recourses and account for competition.

Density effects on the birth rate are modeled according to the Lotka-Volterra assumption of a linear relationship between birth rate and total population density:
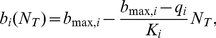
(2)in which b_max, i_ is the birth rate of the i^th^ species without density effects (i.e. the intrinsic birth rate), and K_i_ is the carrying capacity. Note that no competition coefficients are included, so the effect of a competitor on the birth rate is the same as that of a conspecific. In other words, the two species have completely overlapping niches. Eq. 2 gives b_i_(K_i_) = q_i_, so that growth rate of the i-th species in absence of sexual reproduction is zero when N_T_ = K_i_. Moreover, the parameters in the model are time-independent, so that, if found, coexistence is not an effect of environmental fluctuations.

As a conservative approach for species similarity, the parameters of the model b_max,i_ and q_i_, are considered to be equal for both species (hereafter, the species index in these parameters is dropped). By contrast, carrying capacity of Species 2, K_2_, is assumed to be a proportion of the carrying capacity of Species 1, K_1_ (i.e., K_2_ = *β*K_1_ , with 0<*β*≤1). This allows us to introduce an asymmetry in the competitive abilities. As convention, if asymmetry exists, Species 1 is always the best competitor (i.e., *β*<1).

## Results

### Model analysis

**Figure 1:**
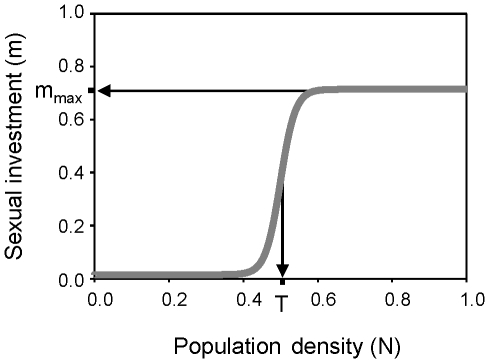
Sexual investment and population density. Relationship between sexual investment (m) and population density (asexual + sexual), as modelled by Eq. 3 (T = 0.5, m_max_ = 0.7 and z = 50).

Density-dependent sexual/diapause investment can be described by a sharp sigmoid function accounting in a continuous fashion for the occurrence of a population growth phase with negligible sexual or diapause investment and a population growth phase with both sexual and asexual reproduction (Fig. 1). An simple instance of such a function for density-dependent sexual investment, m_i_(N_i_) is:
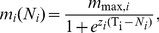
(3)where m_max,i_ is the maximum asymptotic investment in sexual reproduction, T_i_ is the population density threshold for sex induction, defined as the density at which m_i_(N_i_) = m_max,i_/2, and z_i_ is a parameter related to the slope of the response. However, the model resulting from combining Eq. 1 and 3 cannot be analyzed algebraically. Equilibrium analysis for a single species yields transcendental equations, and a Taylor expansion of Eq. 3 truncated to second order is a poor approximation and gives extremely complex equations. This makes it unfeasible to determine the equilibrium values for a two-species system as well as to perform an invasibility analysis, in which the equilibrium density for a system with a single species, the resident, needs to be found.

Alternatively, details of the functional relationship between sexual investment and density can be ignored, while the well-known features (i.e., sex investment determined by density, and sex induced at a density threshold) are taken into account. It can be assumed that, if only one species (the resident) occurs, an equilibrium population density that is greater than zero is achieved, and at that density sexual investment is m_i_*, while sexual investment is m_0_ for the low-density invader species. Therefore, if dA_i_/dt = dS_i_/dt = 0, A_i_>0, and N_j≠i_ = 0, then m_i_(N_i_) = m_i_*. Alternatively, if N_i_→0, then m_i_(N_i_)→m_0_. Using these assumptions, an invasibility analysis is possible.

Two different scenarios are possible: (a) Species 1 (i.e., the superior competitor if an asymmetry exists) as a resident at its single-species equilibrium and Species 2 as invader; and (b) the opposite situation. Densities at equilibrium (A_i_* and S_i_*) for both scenarios were obtained for the resident species. Non trivial solutions for each scenario (Sol. 1a is for the scenario a) are:
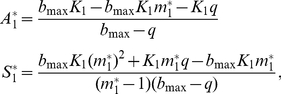
(1a)

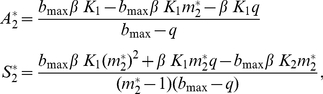
(1b)


Notice that A_i_*>0 needs m_i_*<(b_max_−q)/b_max_. If not, birth rate is overcompensated by the combined effect of investment in sex and mortality.

The per capita rate of increase for the invader species was obtained by equalling the resident species densities to their densities at equilibrium (A_i_*, S_i_*), assuming that the invader was composed exclusively by asexual individuals, and the sex investment of the invader is m_0_. The per capita growth rates corresponding to scenario a and b are respectively (see Appendix A):
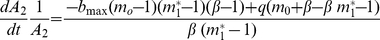
(2a)

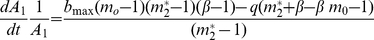
(2b)


As the invasion analysis assumes a low density of invader, no investment in sexual reproduction is likely to happen (m_0_ = 0), consistent with the observation in the wild of a completely asexual phase in cyclical parthenogens. However, at equilibrium density sexual reproduction is expected to occur. From these assumptions (m_i_*>0, m_0_ = 0), the following relationships are found for an invasion to occur (i.e., for (dA_i_/dt)(1/A_i_)>0)

Species 2 is able to invade if
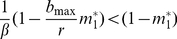
(3a)


Species 1 is able to invade if
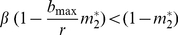
(3b)


**Figure 2:**
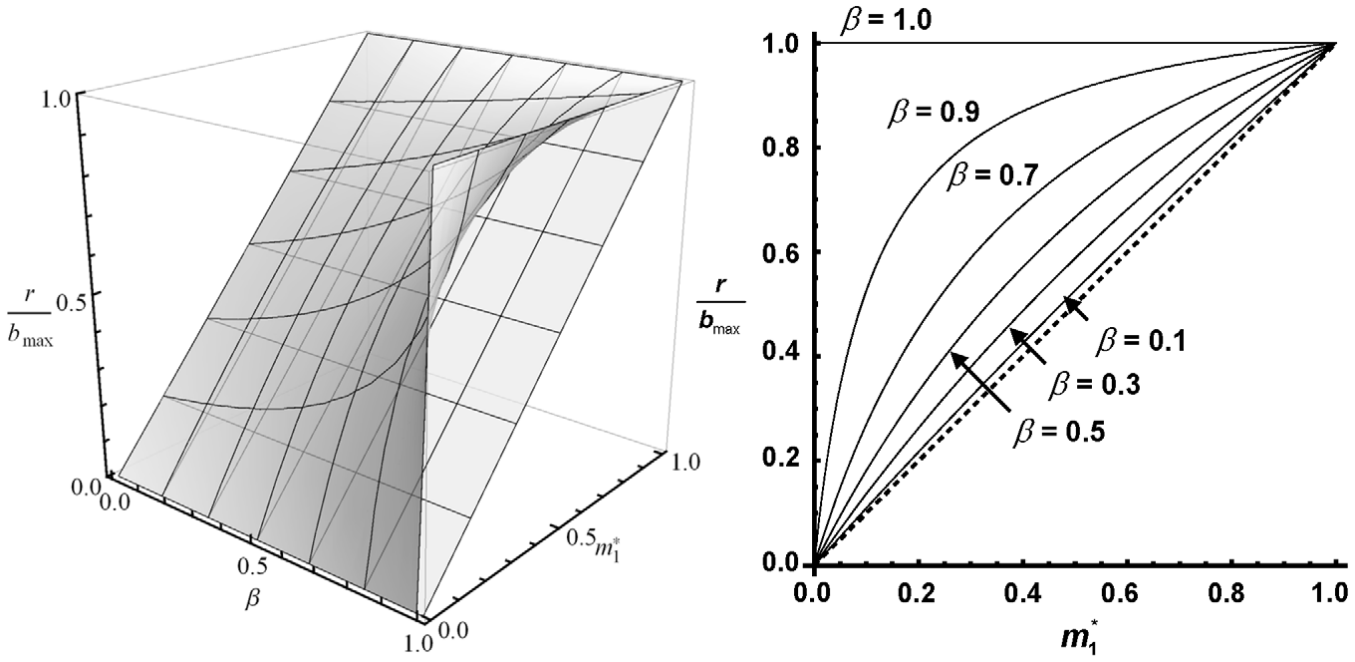
Invasion capability of an inferior competitor having no-sex investment. Invasion capability of an inferior competitor when it is not investing in sex (m_0_ = 0). *Left panel*. Parametric space defined by r/b_max_, the density-dependent sexual investment at equilibrium of the resident species (m_1_^*^) and relative competitive ability of the invader (*β*). The linear surface is defined by the maximum possible sexual investment at equilibrium (m_i_^*^ = r/b_max_), so that the values below that surface do not allow permanence of the resident species. The non-linear surface defines an edge for a positive growth rate of an invader being competitively inferior or equivalent to the resident, so that all the values below that surface imply successful invasion. Hence, all the values between both surfaces allow stable coexistence. *Right panel*. Slides for different values of *β* of the parametric space shown on the left panel. Dotted line shows the maximum possible investment in sex. Values between solid lines and the dotted line are the parametric values allowing stable coexistence.

Here, r = b_max_−q, 0<*β*≤1 and 0<m_i_^*^≤r/b_max_ and these parameters belong to P [0, +∞[. According to Sol. 3b, Species 1 (the superior competitor) is always able to invade. Note that b_max_/r is larger than 1. Sol. 3a shows that the invasion capability of Species 2 depends on the amount of investment in sex of the resident species and the level of competitive asymmetry (Fig. 2). Therefore, Sol. 3a is the condition for reciprocal invasibility and hence for stable coexistence. Accordingly, species with identical competitive abilities (*β* = 1) are able to coexist if some investment in sexual reproduction is made by the resident. Interestingly, as sexual investment increases, higher degrees of asymmetry in competitive abilities are still compatible with stable coexistence.

According to Sol. 3, if no species is investing in sex (m_i_* = m_0_ = 0), invasion capability (dA_i_/dt)(1/A_i_)>0) results in β>1 and 1/*β*>1 for scenario a and b respectively, which never can be accomplished. Thus, Species 2 (i.e. the inferior competitor) is not able to invade in any case. The second condition is always accomplished except for *β* = 1. That is, with no sex, Species 1 will be able to invade the resident population except if it is competitively equivalent. These results are the expected ones under a conventional Lotka-Volterra model for interspecific competition.

**Figure 3:**
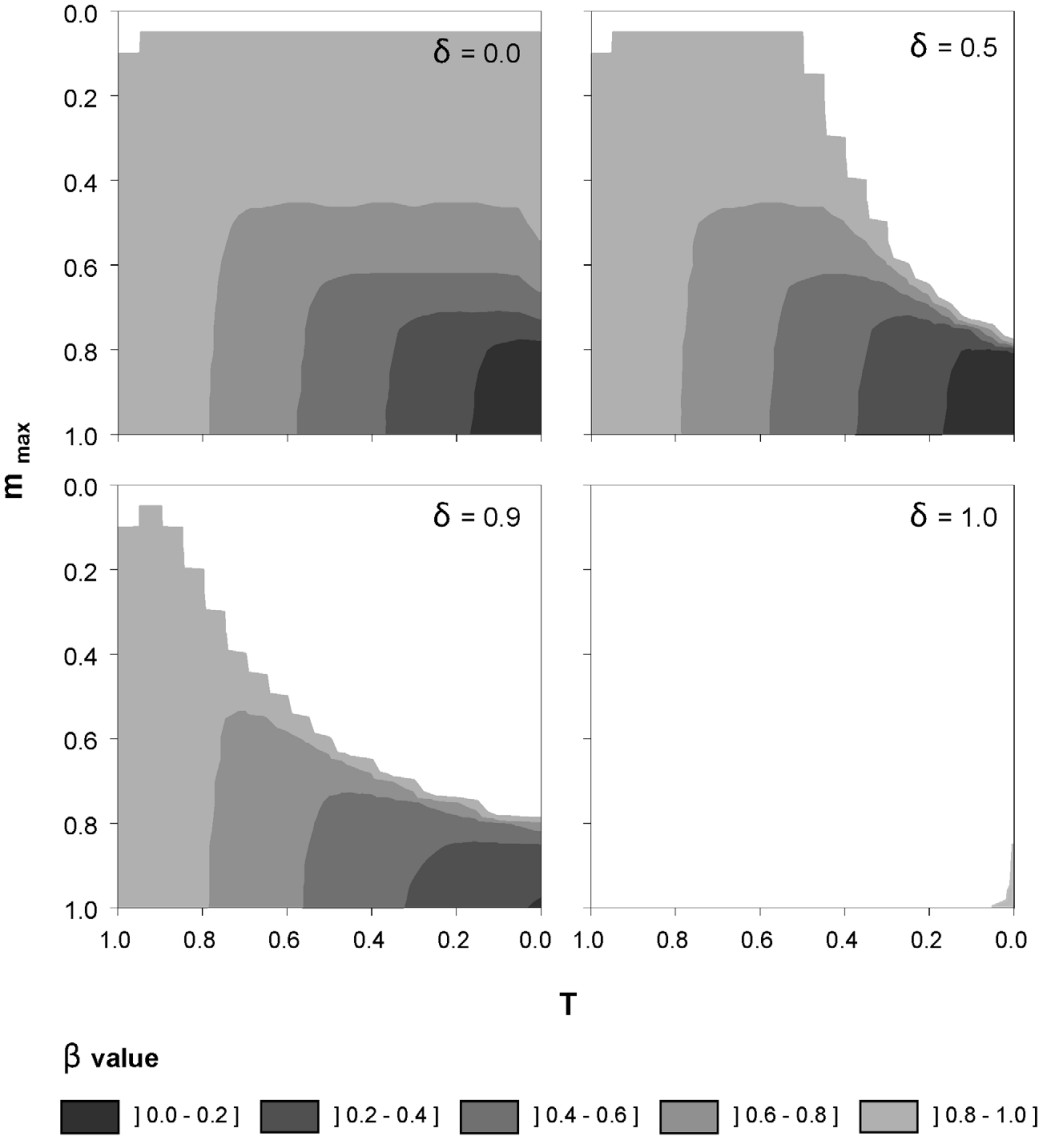
Stable coexistence and heterospecific induction of sex. Stable coexistence of two species with density-dependent investment in sex under different degrees of heterospecific induction of sexual investment (*δ*). Sex investment is defined in terms of T and m_max_. The range of asymmetry in the competition (*β*) that allows coexistence is also shown. Note that no stable coexistence was observed for m_max_ = 0.

As stated in Eq. 1a, our model assumes that the sexual investment of a species is dependent only on the conspecific population density; that is, signal for sex is species-specific. Additionally, we modified the model to allow partial cross induction between species. In this modification, we used Eq. 3 but with the variable N_i_ substituted by N_i_+*δ*N_j_, where *δ* accounts for the similarity between species in their sex-inducing signals (i.e. *δ* = 1 and *δ* = 0 imply respectively complete cross-induction of sex and total specifity of the signal). We explored this scenario by numerical integration. The model was parameterized using a cyclical parthenogenetic rotifer as biological model. K_1_ and b_max_ were rescaled to 1, and q was assumed to be 0.2, which, if b_max_ = 1 d^−1^, gives a maximum population growth of 0.8 d^−1^, a realistic value for rotifers [21]. Several values for two of the three parameters controlling investment in sex, the threshold density (T) and the maximum theoretical investment in sexual reproduction (m_max_), were explored. The other parameter, z, was fixed at a value (50) high enough to cause an almost “on-off” response, in agreement with empirical observations [22]. δ values in the range 0.0–1.0 were tested and we found that stable coexistence, is still possible, although it becomes more unlikely as value of *δ* increases (Fig. 3).

## Discussion

In this paper we identify a heretofore unidentified mechanism that could explain the coexistence of ecologically equivalent species by means of density-dependent sex and/or diapausing investment. In our model, the ultimate reasons for the loss of competitive ability with increasing density are the negative effects of male production and diapause on current population growth. Sexual reproduction and diapause allocates resources that do not translate into current population growth or competition efficiency. Thus, depending on the amount of sex and/or diapause investment, this creates an opportunity for another species to invade a sex and/or diapause-investing resident population, even if the invader is an inferior competitor. This is a novel extension of the conclusions of Zhang and Hanski [8] of how mechanisms based on sexual reproduction could allow the coexistence of ecologically equivalent species without niche differentiation.

It is unlikely that any pair of species can be identical in all of their ecological traits, yet our results for identical species demonstrate that coexistence is possible even in this most stringent case. To date, no study has focused on the effect of density-dependent sex or diapause investment on coexistence. However, Ciros et al. [23] studying the competitive success of three sympatric cryptic cyclical parthenogenetic species from the *Brachionus plicatilis* species complex found a negative relationship between their sexual reproduction investment and competitive success. Our results based on modelling provide guidelines for future empirical studies on competition. For instance, the coexistence of species with no differential predation vulnerability, exploiting a single resource in a constant environment has been shown here to be theoretically possible, and can be tested with simple experiments. We further advocate that studies attributing species coexistence to niche differentiation, also need to consider the possibility of differential sex and diapause investment.

A potential concern about our study is that no species differentiation in the timing of sex would be expected if species niches completely overlap. This concern is based on the assumption that the switch to sexual reproduction occurs when conditions are adverse, e.g., when the density of both the resident and invading species is high, so that birth rate decreases due to competition. This is called the habitat deterioration hypothesis of sex initiation and is only one of several plausible scenarios [24]. For example, species could have evolved different population density signals in allopatry, as a response to physical conditions in their environments. It is known that cryptic rotifer species, which currently coexist, had separate refugia during Pleistocene glaciations [25]. Perhaps of most importance, the timing of sex in facultative sexuals is expected to be shaped not only as a response to anticipated environmental adversity, but by mate encounter probability, which is strictly species-specific. That is, signals for initiating sex are part of quorum sensing mechanism [19]. Because our model deals with real –i.e., reproductively isolated—species, sex induction at low density is not expected to evolve. Otherwise, male-female encounter is unlikely to occur. Moreover, our model suggests this specificity will be evolutionarily stabilized by competition, since that the low density species has invading opportunities by delaying sex (see below).

A complete differentiation in the signals for sex and diapause seems unlikely in the case of closely related species and some level of cross-induction due to heterospecific population density could be expected. For example, in rotifers of the genus *Brachionus* some degree of cross-induction has been reported [20], [26]. Our simulation results also have shown that partial cross-induction still allows coexistence between cyclical parthenogenetic species sharing identical niches. However, an open question is how other density-independent sex or diapause inducing signals would interact with density-dependent sex induction. For example, sexual reproduction in the cladoceran *Daphnia magna* is induced by a suite of factors including crowding, temperature, food level and photoperiod [15]. Some of these factors may exert their effects by altering patterns of temporal niche differentiation.

A second concern about our model is that sex is assumed to make no immediate contribution to current population growth, whereas the diapausing eggs produced sexually could be relevant to coexistence through evolutionary time. However, since sex typically is associated with diapause, sex involves a short-term cost additional to the two-fold cost. This cost results from longer generation times and lowered survival [27]. As a result, sexual offspring are expected to make a negligible contribution to population growth, which is primarily the result of asexual reproduction with short generation times. Of course, these costs should be compensated if, as expected, the life cycle is adaptive. During adverse periods, which are recurrent in many habitats, the active populations disappear, and recolonization relies on the diapausing stages. Then, a longer timescale becomes relevant. However, as long as both species are able to produce viable diapausing eggs, our conclusion on coexistence stands. This is because the invasion process analyzed by our model is merely suspended when habitats are unsuitable, and resumes when conditions favorable for growth return. If invasion is successful in one of the favorable periods, it will be successful the following one as well.

An additional question is how stochasticity interacts with the deterministic dynamics of our models. The coexistence showed by our model is not neutral, and the recovery of rare species found in our analysis is expected to provide some protection against extinction due to random walks. However, the abundance reached by the inferior competitor is relevant to evaluate the effects of demographic stochasticity on random extinction. Nevertheless, at least for some groups where our model is applicable, even low population densities imply large population sizes, making unlikely a strong effect of demographic stochasticity. Moreover, the formation of diapausing banks could buffer against stochasticity, protecting the inferior competitor [7]. Interestingly, weak stochastic effects might suggest that coexistence of ecologically equivalent species might be neutral. Even accepting this hypothesis as plausible, it needs to be contrasted with non-neutral models incorporating relevant lifecycle features of the species involved, and accounting for stable coexistence, as the model developed here.

A question arising from our results is whether the evolution of sexual and/or diapause investment patterns might be evolutionarily shaped by interspecific competition. For instance, a superior competitor would not be invaded by an inferior one if the former is not investing in sex or diapause. However, this investment is necessary to survive through adverse environments or to generate genetic variability. A species that invested less in sexual reproduction and became a better competitor might be compromising its own long-term persistence. Therefore, a trade-off is likely to exist, and an optimal level of sexual and/or diapause investment is expected to evolve. Our results suggest that this optimal level would still mediate coexistence, since coexistence was found with low sex investment. As another example, a highly species-specific response to the sex-inducing signal could be costly (e.g. it could require the maintenance of complex enzymatic machinery to produce the signalling molecule) and be selected against due to this cost. However, it could confer a competitive advantage to an inferior competitor, particularly if the superior competitor has not evolved a species-specific signal. It is an open question if the rates of competitive exclusion would provide time enough for the evolution of differentiation in sexual signals.

The coexistence mechanism identified here could be extended to other species with life cycles where cost of males or cost of diapause are density-dependent (i.e. obligate sexual and obligate asexuals investing in diapause, and facultative sexuals that are not cyclical parthenogens). More generally, density-dependent life-cycle switches –such density-dependent sex or diapause investment–, density-dependent sex ratio mediated by local mate competition [8], [28], and perhaps other density-dependent switches –such as investment in dispersal in aphids [29]– show that plasticity in life-history traits could cause a decrease in growth rates with density, so that coexistence of competitors would be promoted. As life history theory has demonstrated, these traits are evolutionarily shaped by a suite of selective factors including intraspecific relationships, interspecific competition, predation, parasitism and abiotic conditions [30]. Thus, where optimal life-history trait values are not determined uniquely by interspecific competition, competitor coexistence might be possible.
